# Donepezil inhibits neuromuscular junctional acetylcholinesterase and enhances synaptic transmission and function in isolated skeletal muscle

**DOI:** 10.1111/bph.15940

**Published:** 2022-09-15

**Authors:** Robert R. Redman, Harry Mackenzie, Kosala N. Dissanayake, Michael Eddleston, Richard R. Ribchester

**Affiliations:** ^1^ Centre for Discovery Brain Sciences University of Edinburgh Edinburgh UK; ^2^ Pharmacology, Toxicology and Therapeutics, Centre for Cardiovascular Science, Queen's Medical Research Institute University of Edinburgh Edinburgh UK

**Keywords:** acetylcholine, acetylcholinesterase, Alzheimer's disease, anticholinesterase, endplate potential, muscle contraction, neuromuscular block, neuromuscular junction

## Abstract

**Background and Purpose:**

Donepezil, a piperidine inhibitor of acetylcholinesterase (AChE) prescribed for treatment of Alzheimer's disease, has adverse neuromuscular effects in humans, including requirement for higher concentrations of non‐depolarising neuromuscular blockers during surgery. Here, we examined the effects of donepezil on synaptic transmission at neuromuscular junctions (NMJs) in isolated nerve‐muscle preparations from mice.

**Experimental Approach:**

We measured effects of therapeutic concentrations of donepezil (10 nM to 1 μM) on AChE enzymic activity, muscle force responses to repetitive stimulation, and spontaneous and evoked endplate potentials (EPPs) recorded intracellularly from flexor digitorum brevis muscles from CD01 or C57BlWld^S^ mice.

**Key Results:**

Donepezil inhibited muscle AChE with an approximate IC_50_ of 30 nM. Tetanic stimulation in sub‐micromolar concentrations of donepezil prolonged post‐tetanic muscle contractions. Preliminary Fluo4‐imaging indicated an association of these contractions with an increase and slow decay of intracellular Ca^2+^ transients at motor endplates. Donepezil prolonged spontaneous miniature EPP (MEPP) decay time constants by about 65% and extended evoked EPP duration almost threefold. The mean frequency of spontaneous MEPPs was unaffected but the incidence of ‘giant’ MEPPs (gMEPPs), some exceeding 10 mV in amplitude, was increased. Neither mean MEPP amplitude (excluding gMEPPs), mean EPP amplitude, quantal content or synaptic depression during repetitive stimulation were significantly altered by concentrations of donepezil up to 1 μM.

**Conclusion and Implications:**

Adverse neuromuscular signs associated with donepezil therapy, including relative insensitivity to neuromuscular blockers, are probably due to inhibition of AChE at NMJs, prolonging the action of ACh on postsynaptic nicotinic acetylcholine receptors but without substantively impairing evoked ACh release.

**Abbreviations**
AD: Alzheimer's disease
ATH: acetylthiocholine iodide
DTNB: 5,5′‐dithio‐bis (2‐nitrobenzoic acid)
EPC: endplate current
FDB: flexor digitorum brevis muscle
gMEPPs: giant MEPPs
MEPP: miniature EPP
nAChR: nicotinic acetylcholine receptor
NMJ: neuromuscular junction
ROI: region of interest
T50: time to 50% decay
T90: time to 90% decay
TOF: train of four
μ‐CTXGIIIB: μ‐conotoxin GIIIB

**What is already known**

- Some patients taking donepezil, a treatment for Alzheimer's disease, show signs of impaired neuromuscular function.

**What does this study add**

- Donepezil inhibits muscle acetylcholinesterase, extends tetanic muscle contractions and prolongs endplate potentials in mouse muscle.

**What is the clinical significance**

- The data provide a rationale for management of neuromuscular block in anaesthetised patients prescribed donepezil.

## 1 INTRODUCTION

Synaptic transmission at central and peripheral cholinergic synapses that utilise acetylcholine (ACh) as a neurotransmitter is normally terminated by the enzymic action of acetylcholinesterase (AChE), which catalyses hydrolysis of ACh to choline and acetate (Rotundo, 2020; Taylor & Radić, 1994). Donepezil, also referred to in early publications as ‘E2020’, is a piperidine inhibitor of acetylcholinesterase (anti‐AChE) that has been widely prescribed for mild to moderate dementia in Alzheimer's disease (AD). It mitigates cognitive impairment, especially when deployed at early stages of AD (Birks & Harvey, 2006; Dooley & Lamb, 2000; Zhang et al., 2020). Dosage is normally between 5 and 10 mg daily, achieving plasma concentrations in the range of 10–100 nM (Rogers et al., 1998; Rogers & Friedhoff, 1998). However, several clinical case studies have reported adverse signs or symptoms associated with donepezil treatment, consistent with neuromuscular synaptic dysfunction. For instance, about 7% of AD patients taking donepezil experience muscle cramps (Birks & Harvey, 2006; Román & Rogers, 2004). In addition, patients receiving donepezil who undergo surgical procedures for unrelated conditions show enhanced neuromuscular block when depolarising antagonists of nicotinic acetylcholine receptors (nAChRs) such as suxamethonium are administered (Crowe & Collins, 2003; Sprung et al., 1998). Conversely, higher concentrations of non‐depolarising neuromuscular blockers may be required to achieve prolonged muscle relaxation in anaesthetised patients, who are already receiving donepezil (Baruah et al., 2008; Bhardwaj et al., 2011; Jang et al., 2020).

AChE is a powerful enzyme with a catalytic conversion‐to‐affinity ratio (k_cat_/K_m_) equal to about 2 × 10^8^ mol·s^−1^ (Quinn, 1987). At neuromuscular junctions (NMJs) in skeletal muscle, ACh is inactivated in the synaptic cleft within about a millisecond of the synchronous vesicular release of hundreds of thousands of molecules from motor nerve terminals, thereby terminating the activation of postsynaptic nAChRs (Hartzell et al., 1976; Katz, 1996; Katz & Miledi, 1973). Inhibition of AChE, either pharmacologically or by mutation of crucial residues in its protein structure, has marked effects on the persistence of ACh in the synaptic cleft, prolonging endplate depolarisation following its spontaneous or evoked release (Braga et al., 1991; Fatt & Katz, 1952; Maselli & Leung, 1993; Minic et al., 2003). In the absence of significant AChE activity, binding of ACh to nAChRs and diffusion of ACh from the synaptic cleft become the main factors limiting the duration of ACh action (Katz & Miledi, 1973).

Prolonging activation of nAChRs by inhibiting AChE can be directly beneficial in some circumstances. For instance, inhibition of AChEs by carbamates is an established treatment for myasthenia gravis and several other myasthenic syndromes in which nAChR function is compromised (Spillane et al., 2010; Verschuuren et al., 2016). But, in other contexts, anti‐AChEs may have extreme adverse effects. For example, exposure to organophosphorus anti‐AChE compounds is potentially lethal (Chai et al., 2018; Dissanayake, Chou, et al., 2021; Gunnell et al., 2007) and even sub‐lethal concentrations of these compounds have potent effects on physiological properties of NMJs. These include localised contractions of motor endplates, which have been associated with subsequent development of myopathy, nerve terminal degeneration and muscle denervation (Ferry & Cullen, 1991; Leonard & Salpeter, 1979; Meshul et al., 1985; Salpeter et al., 1979).

Adverse signs noted in case reports of patients receiving donepezil are commensurate with pathophysiological effects of donepezil at NMJs, similar to the pharmacological and toxicological effects of other AChE inhibitors. However, whereas there are numerous research reports on the actions of carbamate or organophosphorus inhibitors of AChE at NMJs, there have been few studies of the actions of donepezil on neuromuscular synaptic transmission or function. For instance, donepezil inhibited rat muscle AChE (Kosasa, Kuriya, Matsui, & Yamanishi, 1999) but the IC_50_ was not measured. Donepezil reversed vecuronium block of contractions in isolated guinea pig hemidiaphragm nerve‐muscle preparations with an EC_50_ of 43 nM (Clark et al., 2002), but the methodology was not described. Micromolar concentrations of donepezil enhanced and prolonged spontaneous miniature endplate potentials (MEPPs) in isolated mouse phrenic nerve‐hemidiaphragm preparations (Lin et al., 1996, 1997), but the EC_50_ was not measured. Evoked vesicular release of ACh, based on estimation of the quantal content of endplate potentials (EPPs), indicated no presynaptic effect (Lin et al., 1997), but the measurements were made in solutions containing low Ca^2+^ concentrations that disproportionately reduce mean quantal content (Dodge & Rahamimoff, 1967; Hubbard et al., 1968).

Here, we have re‐examined the hypothesis that the adverse neuromuscular signs associated with administration of donepezil are a consequence of peripheral actions of this compound. We found that the biochemical effect of donepezil in the therapeutic range on AChE enzymic activity was lower in mouse skeletal muscle than in mouse brain but, nevertheless, sufficient to give rise to protracted muscle force responses and prolonged synaptic potentials at NMJs.

## 2 METHODS

### 2.1 Ethics, animals and tissues

Guidelines for the reporting of experiments involving animals (ARRIVE 2.0) were consulted and cross‐checked (https://arriveguidelines.org/arrive-guidelines). The study was designed to measure responses of isolated tissue to administration of compounds in vitro using biochemical assays, mechanical function, electrophysiological analysis and optical measurements using physiological indicators. The animals used in this study were not intentionally randomised, and experimental investigators were not blinded to the nature or concentration of drugs or other chemicals added to the bathing media. Other criteria listed as Essential or Recommended in the ARRIVE 2.0 guidelines are noted in the relevant sections of Methods and Results.

Experiments were carried out on isolated tissues from adult (age 1–12 months) wild‐type CD01 or C57BlWld^S^ mice, whose NMJ function resembles those in human skeletal muscle, specifically with respect to electrophysiological responses of motor endplates to release of ACh from motor nerve terminals in response to single or repeated nerve stimulation (Dissanayake, Margetiny, et al., 2021; Slater et al., 1992). Mice were bred and maintained in University of Edinburgh animal care facilities, under standard conditions closely monitored by appointed Veterinary Officers and regularly inspected under institutional licence by the UK Home Office. Animal studies are reported in compliance with the ARRIVE guidelines (Percie du Sert et al., 2020) and with the recommendations made by the *British Journal of Pharmacology* (Lilley et al., 2020).

Mice of both sexes were killed by isoflurane anaesthetic overdose (>5% in air) and cervical dislocation, in accordance with the UK Home Office Schedule 1. Brains were removed and flexor digitorum brevis (FDB) muscles with attached nerve supplies were rapidly dissected and immersed in mammalian physiological saline (MPS) of composition (mM): NaCl (158), KCl (5), CaCl_2_ (2), MgCl_2_ (1), glucose (5), HEPES (5), adjusted to pH 7.2–7.4 with NaOH (1 M). Solutions were bubbled with room air for at least 20 min. Experiments were conducted at room temperature (18–23°C). The FDB muscle was chosen for this study because its muscle fibres are less than 1 mm in length and thus isopotential with respect to their synaptic responses (Bekoff & Betz, 1977; Ribchester et al., 2004).

### 2.2 Acetylcholinesterase assay

AChE activity was assayed as described previously (Dissanayake, Chou, et al., 2021). Briefly, single brains, or distal hind limb muscles from 2 to 5 mice, were dissected in 0.1 M sodium phosphate buffered saline (PBS), pH 7.4, and homogenised (1 g tissue in 8 ml PBS, pH 7.4) using a Pellet Pestle (Sigma, Z 359971). Quantitative measurements of AChE enzymic activity were made using a modified Ellman method (Ellman et al., 1961; Rosenfeld et al., 2001). Stock solutions were acetylthiocholine iodide (ATH; 1.7397 mg·ml^−1^ in PBS), used as the enzyme substrate, and 5,5′‐dithio‐bis (2‐nitrobenzoic acid) (DTNB; 0.7872 mg·ml^−1^ in 0.2 M PBS, pH 7.4). Muscle or brain homogenate was aliquoted into each well of a 96‐well plate, then an amount of test compound (either donepezil or neostigmine) was added and the volume made up to 200 μl with PBS and incubated for 20 min at room temperature. DTNB (50 μl from stock) was added, followed by ATH substrate solution (50 μl). Triplicate measurement of absorption at 450 nm began immediately and was monitored over time using an MRX microplate reader (Dynex Technologies, Chantilly, USA), and the measurements averaged to give a single reading in each case. Thiocholine production in the test wells was expressed as nmol generated per minute, calibrated with reference to the absorbance change using glutathione as the DTNB reactant, over the range of concentrations that gave a linear response (Eyer et al., 2003).

### 2.3 Muscle force recording

Tibial nerve‐FDB muscle preparations were pinned by their distal tendons to a Sylgard lined chamber, and the proximal tendons were connected by 6/0 silk suture to an MLT0202 force transducer (AD Instruments, Oxford, UK). The preparations were bathed in 10 ml of MPS, and the distal stump of the tibial nerve supply was aspirated into a glass suction electrode. Nerve stimuli (nominally up to 10 V, 0.1–0.2 ms duration, 2–20 Hz) were delivered via a DS2 stimulator (Digitimer, Welwyn Garden City, UK) triggered by a Powerlab 26T interface (AD Instruments). Force recordings were captured via the same interface and digitised at 1 kHz using Labchart 7 software (AD Instruments) running on an Apple iMac computer. Muscle ‘aftercontractions’ (see Results) were quantified using a customised Excel spreadsheet as the fraction of the total area under the curve (AUC) of active tension generation occurring after the end of the nerve stimulus train, as described previously (Dissanayake, Chou, et al., 2021).

### 2.4 Ca^2+^ imaging

FDB muscles were loaded with the acetoxymethylester Fluo4‐AM by incubating them in the dye for 30–45 min. In our hands, standard recommended concentrations of Fluo4‐AM (1–5 μM) did not produce significant labelling of muscle fibres. However, a relatively high concentration of Fluo4‐AM (20 μM), suggested by Professor Richard Robitaille (personal communications), usually achieved adequate loading of a few to several FDB muscle fibres. After labelling, preparations were washed with MPS for 10–20 min and then mounted on the stage of an Olympus BX50WI microscope and imaged using an OptiMOS 2.1MP camera (Photometrics, Newcastle, UK). Images were acquired before and after adding donepezil (1 μM) at 50–100 frames per second (fps), using Micromanager public domain software (Edelstein et al., 2014) (downloadable from https://micro-manager.org/). Images were analysed using ImageJ (downloadable from https://imagej.nih.gov/ij/) or FiJi (downloadable from https://fiji.sc/). Muscle movements in the x,y plane were compensated using the StackReg/TurboReg plugin (downloadable from http://bigwww.epfl.ch/thevenaz/stackreg/). Image stacks of the junctional and extrajunctional regions of NMJs were then analysed using the Z‐axis Profile tool in ImageJ/FiJi, applied to elliptical regions of interest (ROIs) within the selected NMJs or in adjacent regions located more than 100 μm from the middle of an NMJ. Changes in fluorescence intensity were expressed as ΔF/F_o_, where ΔF represented the difference between the ROI intensity and average intensity in the ROI before stimulation (F_o_).

### 2.5 Intracellular recording

Recordings were made from isolated tibial nerve‐FDB muscle preparations pinned to the base of a Sylgard‐lined recording chamber and bathed in MPS. Microelectrodes were pulled with a Flaming‐Brown P87 puller (Sutter Instruments, Novato, CA, USA). Electrodes had resistances typically between 20 and 35 MΩ when backfilled with 3 M KCl. The tibial nerve stump was aspirated into a glass suction electrode connected to a Digitimer DS2 stimulator controlled by either a Digitimer D4030 programmer or a CED Micro1401 interface (Cambridge Electronic Designs, Cambridge, UK) connected to a PC running WinWCP software (Strathclyde Electrophysiology Software, Glasgow, UK; downloadable from https://spider.science.strath.ac.uk/sipbs/software_ses.htm). The isopotential nature of FDB muscle fibres enabled nerve‐evoked EPPs and spontaneous MEPPs to be recorded with high fidelity wherever the tip of the penetrating microelectrode is positioned in the muscle fibre (Ribchester et al., 2004). Muscle fibre action potentials and muscle contractions were blocked by incubating the isolated preparations for 20–30 min in 1–2 μM μ‐conotoxin GIIIB (μ‐CTXGIIIB). Surface muscle fibres were impaled and EPPs and MEPPs captured using an Axoclamp‐2B amplifier (Molecular Devices, Sunnyvale, USA), low‐pass filtered at 3 kHz (Neurolog, Digitimer) and digitised using a CED micro1401 interface connected to a laboratory PC. Residual mains interference was selectively eliminated using a Humbug filter (Digitimer). Muscle fibre recordings with initial resting membrane potentials less negative than −50 mV or those in which membranes depolarised by more than 20 mV during recording were discarded. Recordings were made from 5 to 15 muscle fibres in MPS and then after adding donepezil at concentrations ranging from 10 nm to 10 μM. For each fibre impaled, MEPPs were first registered in 30 s recordings, without nerve stimulation. Then, normally in the same muscle fibre, the nerve was stimulated supramaximally (1–10 V) for 30 s at 1 Hz. EPPs and MEPPs were analysed using WinWCP and Minianalysis (Synaptosoft, Atlanta, USA) software, respectively. MEPPs were aligned by their 50% time to peak then averaged. Single exponential time constants were calculated from best least squares fits to the 10%–90% decay of these averaged MEPPs. ‘Giant’ MEPPs (gMEPPs), defined here as spontaneous events that were more than 2.5 times larger than the median MEPP amplitude (Gundersen, 1990), were excluded from the analysis. WinWCP was used to measure peak amplitude, rise time, half‐decay time (T50) and time to 90% decay (T90) of EPPs. The recorded amplitudes of EPPs were compensated for non‐linear summation using the McLachlan–Martin formula: *EPP′ = EPP*_*O*_ */(1 − f·EPP*_*O*_ *)/(|E*_*M*_ *− E*_*R*_ *|)*, where the compensated EPP amplitude (*EPP′*) was derived from the observed amplitude (*EPP*_*O*_) and the driving force: that is, the difference between the observed resting membrane potential (*E*_*M*_) and the transmitter null (reversal) potential (*E*_*R*_), assumed here to be −5 mV. The value of the McLachlan–Martin variable factor (*f*) was taken to be 0.5 (McLachlan & Martin, 1981). EPP amplitudes were then further corrected to a resting potential of −70 mV using the formula: *EPP*_*C*_ *= EPP′·(70 − E*_*R*_ *)/(|E*_*M*_ *− E*_*R|*_ *)*, where *EPP*_*C*_ was the corrected EPP amplitude.

For quantal analysis, individual MEPP amplitudes (excluding gMEPPs) recorded in each fibre were corrected to −70 mV before calculating the mean amplitude. Quantal content was calculated from the ratio of the corrected mean EPP amplitude to the corrected mean MEPP amplitude. Synaptic depression (*D*_*m*_) was calculated in two ways: first, by analogy with train‐of‐four (TOF) measurements in tension recordings, from the ratio of the corrected EPP amplitudes of the fourth and first EPPs recorded at 1 Hz *(%D*_*TOF*_ *= 100·(1 − EPP*_*4*_ */EPP*_*1*_ *))*; and, second, from the ratio of mean quantal content of the first four EPPs (*m*_*1–4*_) to the mean of the quantal contents of the 10th EPP to the last in the train (*m*_*10+*_): *%D*_*m*_ *= 100·(1 − [m*_*1–4*_ */m*_*10+*_ *])*.

### 2.6 Data and statistical analysis

The data and statistical analysis comply with the recommendations on experimental design and analysis in pharmacology, as set out in the relevant Editorial in the *British Journal of Pharmacology* (Curtis et al., 2018). Data were analysed statistically using Prism 7 (GraphPad, San Diego, CA, USA). The data were not discriminated with respect to sex or age of the mice. With reference to sampling and statistical testing, ‘n’ refers to the number of muscle fibres sampled and N refers to the number of mice from which FDB nerve‐muscle preparations were made. Graphs indicate means with either standard deviation (SD) of measurements from individual muscle fibres and either SEM or 95% confidence intervals (CIs) when data were summarised with respect to mice. Other distributions of data values are indicated by medians, interquartile ranges (IQRs) and 5%–95% limits. Non‐linear least squares concentration–response curves were fitted using four‐parameter logistic regression (4PL) using routines built into GraphPad Prism 7. Student's *t* tests or ANOVA were used to assess significance of mean differences of continuous parametric data between two groups or multiple groups, respectively, assuming Gaussian distributions in the test and control groups, and in those instances when ANOVA indicated significant differences between groups (*P* < 0.05), applying Tukey's or Dunnett's post hoc evaluations, where appropriate. Chi‐squared tests were used to evaluate significance of differences in the incidence of gMEPPs. The Mann–Whitney *U*‐tests, Wilcoxon signed rank tests or Kruskal–Wallis tests were used to assess non‐parametric data or data for which Gaussian distributions were not assumed. Dunn's post hoc test was used for comparison of means in cases where the Kruskal–Wallis tests indicated a significant difference (*P* < 0.05) between multiple groups. The number of animals (N) was taken to be the independent variable for calculation of significance at α = 0.05. Post hoc power calculations of Type 2 error probability (β) or recommended sample sizes were made using an online calculator (https://www.stat.ubc.ca/~rollin/stats/ssize/n2.html).

### 2.7 Materials

Sigma‐Aldrich (Gillingham, UK) supplied NaCl, KCl, CaCl_2_, MgCl_2_, glucose, HEPES, NaOH, NaH_2_PO_4_, Na_2_HPO_4_, ATH, glutathione, donepezil and neostigmine. Stock solutions of neostigmine (1–10 mM) or donepezil (1 mM) in water or PBS were diluted with MPS. DTNB and Fluo‐4AM were supplied by ThermoFisher Scientific (Glasgow, UK). Bachem (Bubendorf, Switzerland) supplied μ‐CTXGIIIB, and the microelectrode glass capillary was supplied by Harvard Apparatus (Cambridge, UK).

### 2.8 Nomenclature of targets and ligands

Key protein targets and ligands in this article are hyperlinked to corresponding entries in http://www.guidetopharmacology.org and are permanently archived in the Concise Guide to Pharmacology 2021/22 (Alexander, Fabbro, et al., 2021; Alexander, Kelly, et al., 2021; Alexander, Mathie, et al., 2021).

## 3 RESULTS

### 3.1 Donepezil inhibits muscle AChE and antagonises non‐depolarising neuromuscular block

**FIGURE 1:**
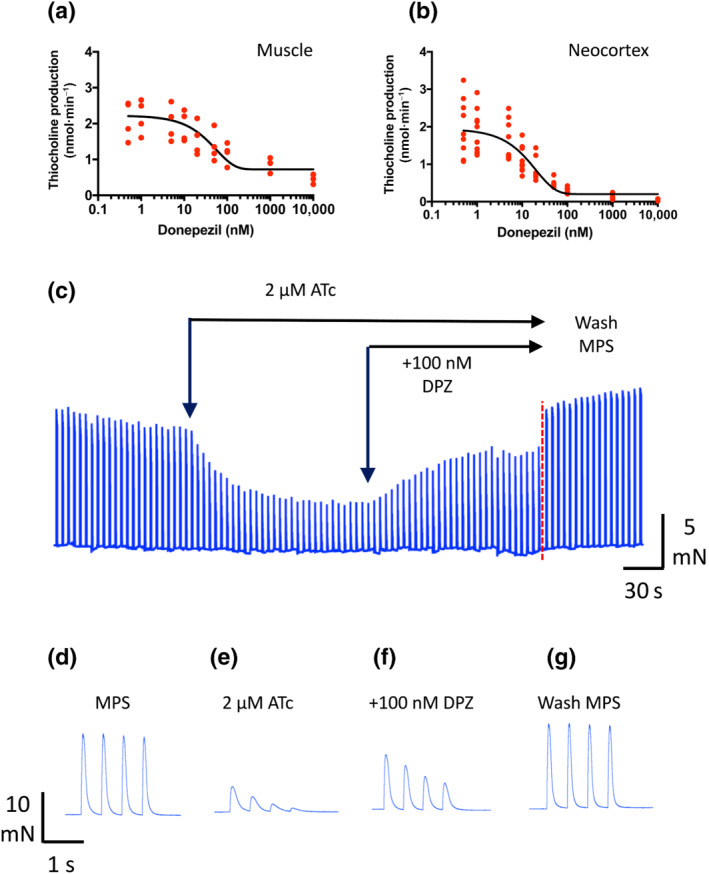
Donepezil inhibits muscle acetylcholinesterase (AChE) and antagonises non‐depolarising neuromuscular block. (a) Inhibition of mouse muscle AChE activity with increasing concentration of donepezil based on thiocholine production (see Methods). Each data point is the average of triplicate measurements from one homogenate prepared from 2 to 5 mice. The IC_50_ based on the sigmoidal best fit to the data was estimated to be about 30–50 nM but inhibition was evidently incomplete up to concentrations of 10 μM. (b) Similar analysis from homogenates of mouse neocortex (mean of triplicate measurements of one mouse brain per point). The IC_50_ was about 10–20 nM and inhibition was virtually complete in 100 nM donepezil. (c) Continuous recording of flexor digitorum brevis (FDB) muscle twitch amplitudes before and after adding a submaximal blocking dose of atracurium (ATc; 2 μM) to the recording chamber. Donepezil (100 nM, DPZ) partly antagonised the partial neuromuscular block. Twitch tension was restored after washing both compounds from the recording chamber with normal mammalian physiological saline (MPS). (d–g) Trains‐of‐four twitch responses from the recording shown in (c) on an expanded time scale; in MPS (d), after adding atracurium (e), following further addition of donepezil (f) and after restoring normal MPS (g).

The IC_50_ for inhibition of purified human AChE by donepezil is about 18 nM (Clark et al., 2002). Here, we found that donepezil inhibited AChE enzymic activity in mouse hind limb muscle homogenates with an approximate IC_50_ of 30–50 nM, based on the best sigmoidal curve fit to the data (Figure 1a), but inhibition appeared incomplete even at a concentration of 10 μM. By comparison, our estimate of the IC_50_ for donepezil using brain (neocortical) homogenates was about 10–20 nM and inhibition appeared complete at a concentration of 100 nM (Figure 1b). The IC_50_ for inhibition of muscle AChE activity by neostigmine, using the same method, was also about 10–20 nM (Figure S1). Thus, donepezil appears to have a lower efficacy in inhibiting mouse muscle AChE compared with AChE in neocortex, or compared with inhibition of murine muscle AChE by the carbamate anti‐AChE, neostigmine.

An EC_50_ of 43 nM was reported previously for antagonism of neuromuscular block by donepezil in isolated guinea pig diaphragm preparations, although the protocol was not described or illustrated in that study (Clark et al., 2002). Figure 1c shows confirmation of the functional effect of 100 nM donepezil in an isolated mouse tibial nerve‐FDB muscle preparation. Stimuli were delivered to the tibial nerve in TOFs at 2 Hz, every 10 s. In normal MPS, this produced twitch contractions that varied little during each TOF (Figure 1d). As expected, twitch amplitude decreased and TOF fade increased after adding the non‐depolarising nAChR antagonist atracurium (2 μM; Figure 1e). Twitch attenuation and fade partly recovered, by more than 50%, after adding 100 nM donepezil (Figure 1f). Washing both compounds from the recording chamber restored normal twitch contractions (Figure 1g). Although we did not determine an EC_50_ for donepezil in these experiments, qualitatively the characteristic pattern of reversible twitch response shown in Figure 1c–g mimics what has been well documented in similar experiments, for example, with carbamate anticholinesterases, such as neostigmine (Braga et al., 1993).

### 3.2 Donepezil prolongs tetanic muscle force

Previous studies have shown that blocking AChE with carbamate or organophosphorus anti‐AChEs gives rise to prolonged post‐tetanic muscle contractions (resembling muscle cramps) that sometimes persist for several seconds after delivery of brief tetanic nerve stimulation (Dissanayake, Chou, et al., 2021; Hong & Chang, 1993). These post‐tetanic muscle responses are associated with sustained, slowly relaxing contractions localised to NMJs (Burd & Ferry, 1987; Dissanayake, Chou, et al., 2021; Ferry & Cullen, 1991).

**FIGURE 2:**
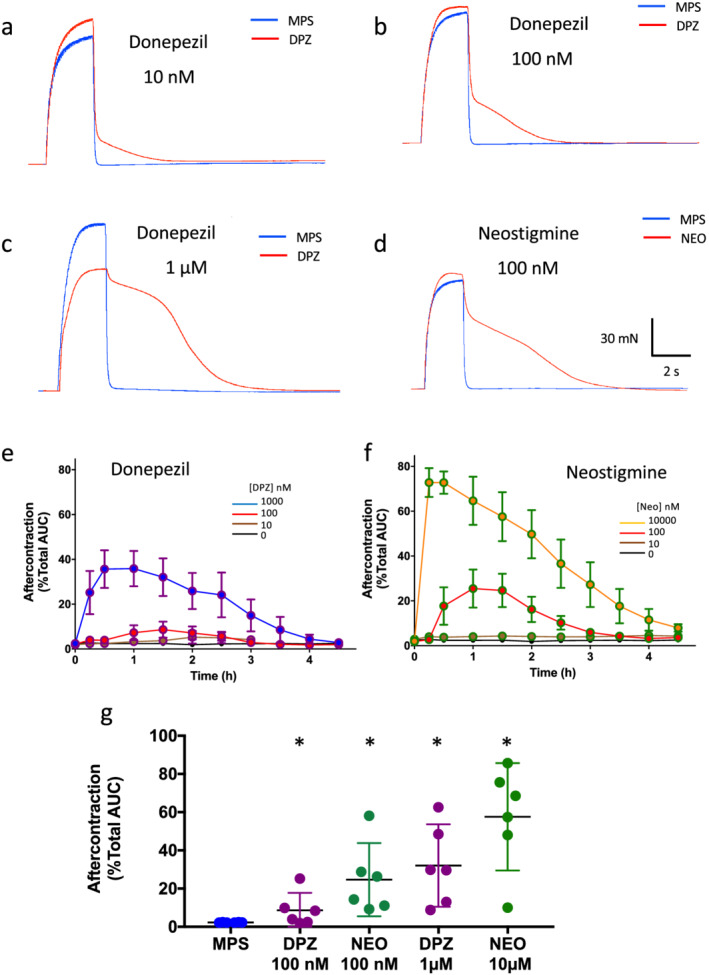
Donepezil prolongs tetanic muscle force. (a–c) Isometric tension recordings from isolated flexor digitorum brevis (FDB) muscles during repetitive stimulation of the tibial nerve supply for 2 s at 20 Hz. Each panel shows the tension response in mammalian physiological saline (MPS) and 1–1.5 h after adding donepezil (DPZ) at the concentrations indicated. In control solution, tetanic muscle responses relaxed promptly at the end of the stimulus train but relaxation was progressively delayed (‘aftercontraction’) after adding donepezil. (d) Neostigmine (NEO; 100 nM), a carbamate anti‐AChE, had similar effects on relaxation of muscle tetani evoked by tibial nerve stimulation. (e, f) Time course of development and decay of aftercontractions after adding donepezil (e) or neostigmine (f) at the range of concentrations indicated, expressed as a percentage of the total area under the curve (AUC) during and after tetanic stimulation. Each point represents the mean ± SEM of recordings from 5 to 6 mice. The lowest to highest lines represent data in MPS (0), 10 nM, 100 nM and 1 μM donepezil (e), or MPS, 10 nM, 100 nM and 10 μM neostigmine (f). (g) Data summarising fractional AUC measurements for aftercontractions recorded 1 h after adding either donepezil or neostigmine at the concentrations indicated. Each point represents data from one nerve‐muscle preparation (N = 5–6 mice). Bars indicate mean ± 95% confidence intervals. *P < 0.05, significantly different from MPS; paired Wilcoxon test.

Figure 2a–c shows examples of tetanic muscle force generation that occurred when tibial nerve‐FDB muscle preparations were stimulated at 20 Hz, before and after adding donepezil (10 nM to 1 μM), which resulted in a concentration‐dependent increase in the magnitude of ‘aftercontractions’, lasting several seconds after the initial tetanic responses. For comparison, Figure 5d shows a qualitatively similar but larger effect of the carbamate anti‐AChE neostigmine (100 nM). The aftercontractions, expressed as a percentage of the total area under the muscle tetanic force–duration curve, became evident 10–45 min after adding donepezil or neostigmine, respectively, to the recording chamber and reached maximum levels 0.5–2 h later, then declined (Figure 2e,f). Data summarising measurements of area under the tension–time curve are shown in Figure 2g. For instance, within 1.5 h of adding donepezil (100 nM), post‐tetanic force–duration increased by about a factor of 3 and in 1 μM donepezil, the median post‐tetanic area under the force–duration curve increased about 10‐fold. However, 100 nM neostigmine caused about a 20‐fold increase in the duration of aftercontractions.

As expected, endplate contractions underlying muscle aftercontractions were associated with sustained increases in endplate Ca^2+^, confirming evidence obtained more indirectly from effects of other anti‐AChE compounds (Dissanayake et al., 2022; Ferry & Cullen, 1991; Leonard & Salpeter, 1979). Optical measurements of fluorescence at motor endplates after adding donepezil were technically challenging because muscle contraction in response to tibial nerve stimulation still occurred in the presence of the toxin μ‐CTXGIIIB, as when AChE was inhibited either with donepezil or other anti‐AChEs (Dissanayake, Chou, et al., 2021). Three‐dimensional (x,y,z) displacement of the endplate region from the microscopic plane of focus made accurate measurements of endplate fluorescence almost impossible under these experimental conditions. However, in one imaging experiment, the endplate contraction produced relatively little displacement in the focal plane (z‐axis) and post hoc digital compensation for lateral (x,y) displacement during stimulation in this instance therefore enabled continuous measurement of endplate fluorescence during and after the stimulus train (see Methods). Ca^2+^ signals in this fibre showed distinctive, prolonged Fluo4 fluorescence localised to the endplate region (Video S1 and Figure S2). No intracellular Ca^2+^ signals were observed at NMJs in control MPS (no donepezil) when muscle membrane excitability and contraction were blocked with μ‐CTXGIIIB.

### 3.3 Donepezil prolongs EPPs

As donepezil inhibited muscle AChE, antagonised neuromuscular block and prolonged muscle tetani, we expected that endplate depolarisation following vesicular release of neurotransmitter (ACh) donepezil would also be prolonged, extending the duration of spontaneous MEPPs and nerve‐evoked EPPs. As indices, we recorded both the decay time constant (τ) of the best single exponential fit to the repolarising phase of MEPPs and the overall duration of EPPs, expressed as the sum of the EPP rise time (5%–95% of peak) and time from peak to 90% decay (T90).

**FIGURE 3:**
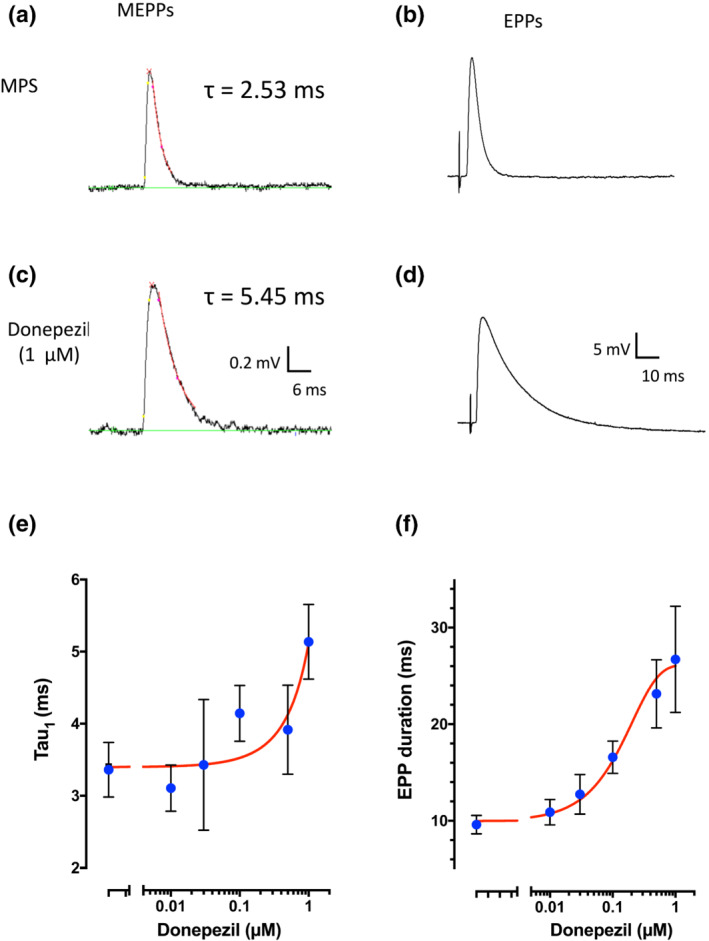
Donepezil prolongs miniature endplate potentials (MEPPs) and endplate potentials (EPPs). (a–d) Examples of averaged MEPPs (a, c) and single EPPs (b, d) from recordings of individual muscle fibres made in control mammalian physiological saline (MPS) and donepezil (1 μM), as indicated. Red superimposed lines in (a, c) indicate non‐linear least squares single exponential fits to the averaged MEPP decays. The single exponential decay time constant (τ) of the averaged MEPP in these two fibres is indicated. (e, f) Donepezil concentration–response data (blue points) and non‐linear least squares fits (red curves) for MEPP decay time constant (g) and EPP duration (h). Each point represents mean data from 9 to 30 muscle fibres (N = 3–5 mice). Error bars are 95% confidence intervals based on numbers of mice.

**FIGURE 4:**
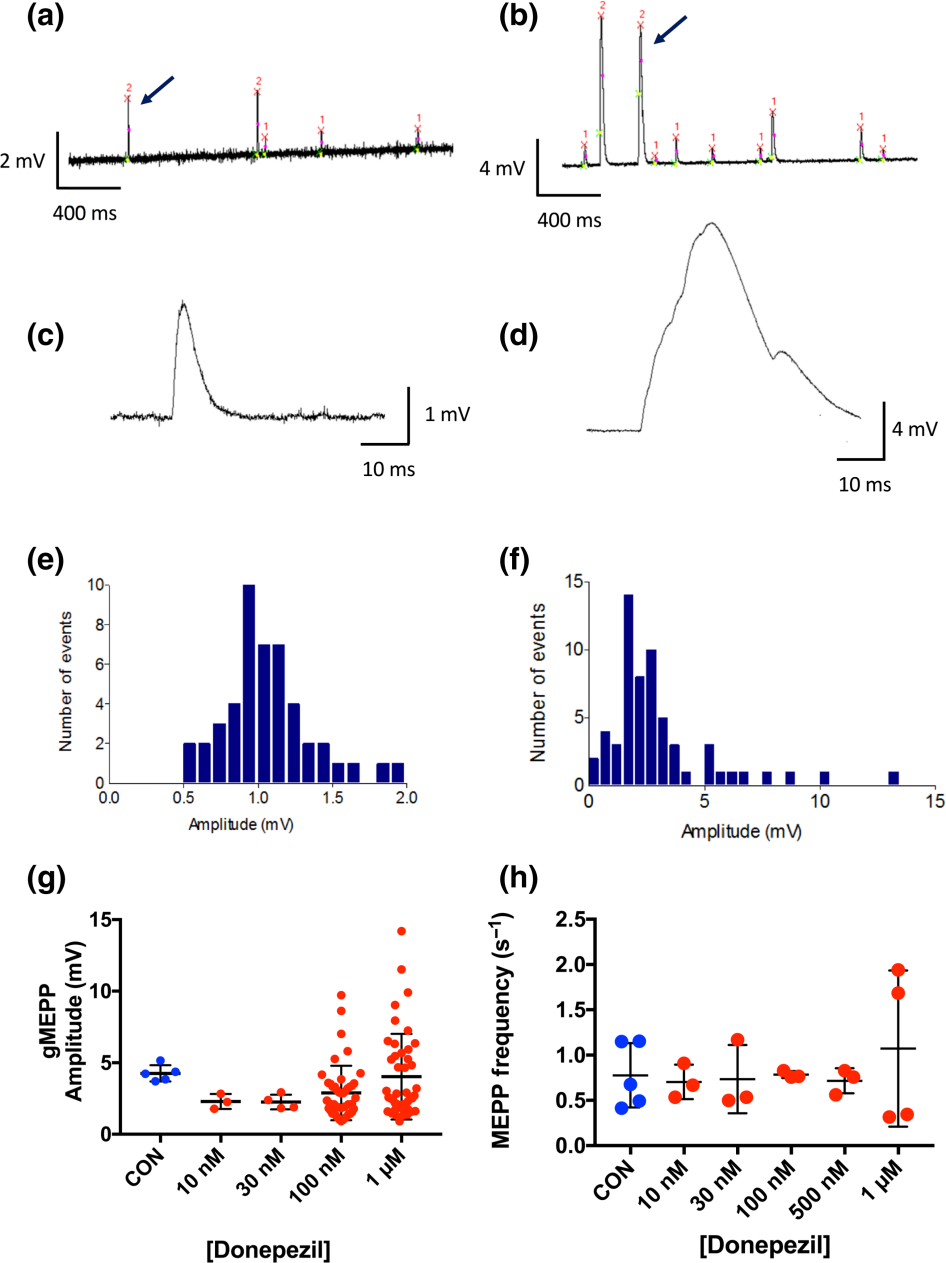
Donepezil increases incidence and magnitude of gMEPPs. (a, b) Slow time‐base recordings of miniature endplate potentials (MEPPs) from a muscle fibre in mammalian physiological saline (MPS) (a) and, in a different fibre, in 1 μM donepezil (b). In both these examples, MEPPs are interspersed with ‘giant’ MEPPs (gMEPPs) more than twice the median MEPP amplitude. Markings in red represent spontaneous (MEPP) events as detected by Minianalysis software. (c, d) Examples of gMEPPs, corresponding to those indicated by arrows in (a, b) and shown on a faster time‐base. The example shown in (d), recorded in 1 μM donepezil, evidently comprises distinctive steps, suggesting spontaneous but desynchronised release of several vesicular quanta of acetylcholine (ACh) from the motor nerve terminal supplying this neuromuscular junction (NMJ). Note differences in voltage calibration in (a, b) and in (c, d). (e, f) MEPP amplitude histograms from the recordings illustrated by traces shown in (a–d), emphasising the magnitude of gMEPPs recorded in donepezil. Note differences in scale of the abscissa in (f). (g) Plot of gMEPP amplitudes recorded in 30 s recordings of spontaneous activity in control MPS (CON) and in increasing concentrations of donepezil. Each point represents one gMEPP and error bars are means ± SD (n = 3–10 muscle fibres per mouse; N = 3–5 mice at each concentration). The largest gMEPPs were observed in 100 nM to 1 μM donepezil. (h) Mean MEPP frequency (excluding gMEPPs) overall was unaffected by increasing concentrations of donepezil. Each point represents the mean frequency based on MEPP recordings from flexor digitorum brevis (FDB) muscles dissected from one mouse and error bars indicate the mean ± SEM of these measurements (n = 3–10 muscle fibres per mouse; N = 3–5 mice).

Figure 3a–d shows examples of averaged MEPPs, based on alignment of spontaneous events occurring in 30 s episodes of recording, in control MPS and in 1 μM donepezil (Figure 4a,c), and individual nerve‐evoked EPPs (Figure 3b,d). Muscle action potentials were blocked in these experiments by preincubating the FDB nerve‐muscle preparations in μ‐CTXGIIIB (see Methods). Summary data (Figure 3e,f) suggested that MEPP decay time constant progressively increased by about 67%, in 1 μM donepezil (Figure 3e). But the increase in nerve‐evoked EPP duration (sum of the rise time and T90 decay) was almost threefold in 1 μM donepezil. Regression analysis based on non‐linear least squares fit indicated a half‐maximal effect (EC_50_) of donepezil on EPP duration at about 114 nM (R^2^ = 0.584, DF = 106; Figure 3f).

Together, these effects are consistent with prolonged activation of nAChRs, limited by diffusion of ACh from the synaptic cleft of NMJs, when enzymic hydrolysis of ACh is inhibited, as deduced for other anti‐AChEs (Fatt & Katz, 1952; Katz & Miledi, 1973).

### 3.4 Donepezil increases incidence and magnitude of gMEPPs

Figure 4 indicates that spontaneous MEPP frequency was unaffected by donepezil (see below) but the incidence and amplitude of aberrant gMEPPs, arbitrarily defined here as spontaneous events more than 2.5 times the magnitude of median MEPP amplitude, were increased. Examples of gMEPPs observed in recordings made in MPS and in donepezil (1 μM) are shown in Figure 4a–d. Contingency analysis of gMEPP incidence, reported in Table S1, showed that the number of fibres expressing at least one gMEPP during 30 s of recording was significantly increased in 1 μM donepezil (χ^2^ = 13.22, chi‐squared test for trend).

The examples shown in Figure 4a–d also illustrate that the largest gMEPPs were seen in recordings made after adding donepezil (10 nM to 1 μM), confirmed by inspection of the amplitude histograms from these representative recordings (Figure 4e,f) and the summary data shown in Figure 4g. Overall, the mean amplitude of gMEPPs recorded in MPS was similar to that in 1 μM donepezil. However, several of the recordings in donepezil (100 nM to 1 μM) contained gMEPPs exceeding 10 mV (Figure 4b,d). Events of this magnitude would likely have been sufficient to exceed the action potential firing threshold in preparations not preincubated in μ‐CTXGIIIB (Dissanayake, Margetiny, et al., 2021; Wood & Slater, 1995), triggering spontaneous muscle contractions. Inspection of some of our muscle tension recordings at high gain provided evidence of spontaneous, weak twitching of muscles incubated in donepezil (10 nM to 1 μM; Figure S3). Spontaneous twitching was not normally observed in preparations bathed in control MPS solution.

**FIGURE 5:**
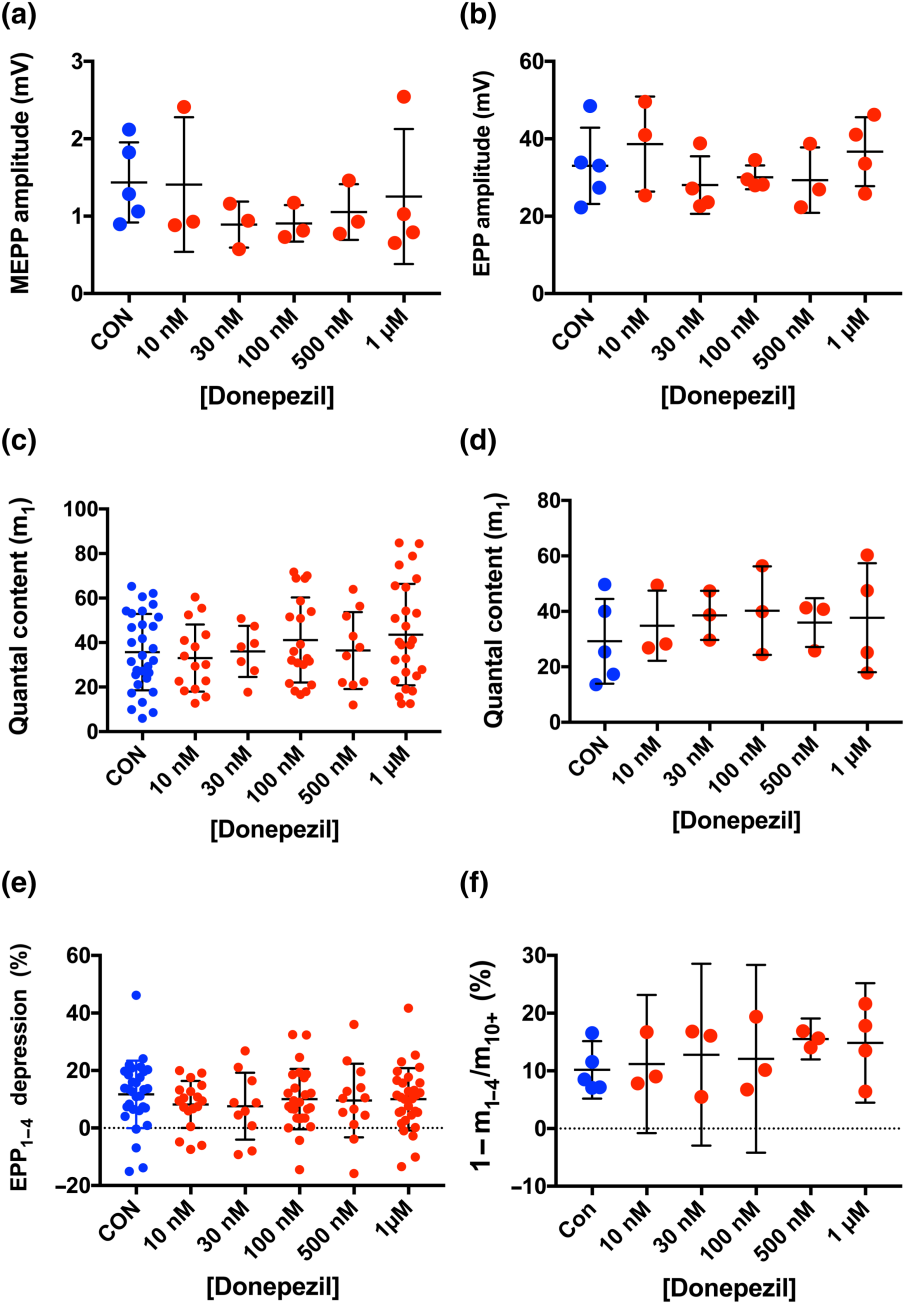
Endplate potential (EPP) quantal content is unimpaired by donepezil. (a) Mean miniature EPP (MEPP) amplitudes, corrected to a resting membrane potential of −70 mV. Each point represents the mean of recordings from 3 to 10 muscle fibres per mouse. There was no significant effect of donepezil over the range of concentrations indicated. (b) Mean amplitudes of the first EPP recorded in trains of 30, evoked at 1 Hz. Each point is the average of recordings from 3 to 10 muscle fibres per mouse, after adjusting EPP amplitudes for non‐linear summation and correcting to a resting membrane potential of −70 mV (see Methods). (c) Quantal content of the first EPP in each recording, obtained directly by dividing corrected EPP amplitude by the corresponding corrected mean MEPP amplitude (excluding giant MEPPs [gMEPPs]). Each point represents the estimate of quantal content from one muscle fibre. Bars represent mean ± SD. (d) Mean quantal content of initial EPPs recorded from preparations dissected from each mouse. Each point represents the mean quantal content from one mouse. There was no significant effect of donepezil on mean quantal content. (e) EPP depression calculated from the ratio of the fourth EPP to the first (EPP_1–4_) in trains of 30 EPPs evoked at 1 Hz. Each point represents data from one muscle fibre. Bars indicate mean ± SD. (f) Depression of EPP quantal content based on ratio of mean quantal contents of the first four EPPs (m_1–4_) to the mean quantal content of EPPs from the 10th to the last EPP (normally the last 20 EPPs) in the train (m_10+_). Each point is the mean depression per mouse based on recordings from 3 to 10 muscle fibres in flexor digitorum brevis (FDB) preparations from one mouse. Error bars show the means of these values with 95% confidence intervals.

Despite the increased incidence of gMEPPs, the mean frequency of all MEPPs, including gMEPPs, were similar in MPS and after adding donepezil (Figure 5f) and any differences were unlikely to be of functional significance. Overall, MEPP frequency varied in control (MPS) solution with an IQR of 0.39–0.90 s^−1^ and in 1 μM donepezil (IQR = 0.42–1.84 s^−1^), which was within the normal range noted in our previous studies of FDB muscles.

### 3.5 EPP quantal content is unimpaired by donepezil

Prolonged postsynaptic activation of AChR would be exacerbated if donepezil also increased neurotransmitter release, as reported for neostigmine and other incidental inhibitors of presynaptic voltage‐sensitive K^+^ conductance (Braga et al., 1991, 1993; Dissanayake, Margetiny, et al., 2021). A previous study, using mouse hemidiaphragm, reported that donepezil increased MEPP amplitude but did not affect quantal content, a measure of the amount of evoked vesicular release of ACh (Lin et al., 1997). However, EPP amplitudes were not reported in that study, and estimates of quantal content were made in a ‘high Mg^2+^/low Ca^2+^ solution’, which substantially reduces mean quantal content. The concentrations of these divalent cations were not specified but were evidently sufficient to cause transmission ‘failures’, allowing quantal content to be estimated without measuring EPP amplitude (Boyd & Martin, 1956). The levels of evoked release in normal divalent cation concentrations are more physiologically and clinically relevant so, here, we measured EPP amplitude and estimated quantal content in physiological solutions containing 2 mM Ca^2+^/1 mM Mg^2+^, approximating to the plasma concentrations of these ions in vivo. Muscle action potentials and twitches were blocked by preincubating the isolated FDB muscles with the selective Na_V_1.4 channel blocker, μ‐CTXGIIIB.

Somewhat to our surprise (as the data appeared to conflict with previous reports), although donepezil prolonged MEPP and EPP decay (see above), it did not enhance mean MEPP amplitude, even at a concentration of 1 μM (Figure 5a). EPP amplitudes, corrected for non‐linear summation and variation in resting potential, were also unaffected by donepezil (Figure 5b), ranging from 22 to 46 mV (IQR) in both control (MPS) solution and 1 μM donepezil. Mean corrected EPP amplitude in these mice was similar to the EPP amplitudes recorded in MPS from FDB muscle fibres in our previous studies (Gillingwater et al., 2002; Ribchester et al., 2004).

The absence of overt effects on either MEPP and EPP amplitudes implies that neither quantal size nor quantal content of evoked vesicular release of ACh were substantively altered by donepezil. Data supporting this conclusion were obtained from stable muscle fibre recordings in which the resting membrane potential drifted by less than 10 mV during the recording of MEPPs and EPPs. We calculated quantal content directly from the corrected EPP/MEPP ratio in these fibres (see Methods). Analysis of recordings from individual muscle fibres (Figure 5c) indicated that quantal contents varied twofold to threefold between muscle fibres. The IQR was 23–52 quanta in control (MPS) solution (n = 30 muscle fibres) compared with an IQR of 22–65 quanta in 1 μM donepezil (n = 26 fibres). Preliminary analysis of data with mice as the independent variable also indicated no statistically significant differences in mean quantal content over the range of donepezil concentrations we tested. Furthermore, these values (Figure 5c) are within the normal range for rodent NMJs (Gillingwater et al., 2002; Wood & Slater, 1997). Post hoc power calculations suggested that, given the amount of variability within and between quantal contents in these groups, a sample size of N = 66 mice in each group would be required to establish whether the mean difference was statistically significant, based on normal threshold criteria (α = 0.05; 1 − β = 0.8). We considered the use of a large number of additional animals required to meet these statistical criteria was not justified, and we therefore accepted the null hypothesis (estimated Type 2 error probability, β = 0.1).

Measurements of synaptic depression during repetitive stimulation supported the EPP quantal analysis and provided another indicator of presynaptic function, as synaptic depression increases as basal quantal content increases (Dissanayake, Margetiny, et al., 2021; Ruiz et al., 2011). We measured synaptic depression here from the ratio of the fourth and the first EPP (EPP_4/1_) in trains evoked at 1 Hz and by comparing the quantal contents of the first four EPPs (m_1–4_) and the 10th to last EPPs (m_10+_) in trains of up to 30 EPPs evoked by stimulation of the tibial nerve at 1 Hz (see Methods).

The data presented in Figure 5e did not indicate an effect of donepezil (10 nM to 1 μM) on EPP depression. Linear regression analysis indicated the effect of donepezil on EPP_4/1_ depression was not statistically significant (R^2^ = 0.645). Analysis of variance also did not indicate any significant effect of donepezil on the m_10+_/m_1–4_ quantal content ratio. Post hoc power analysis indicated that a sample size of approximately N = 19 mice per group would have been required to evaluate whether the small difference in mean m_10+_/m_1–4_ depression in 1 μM donepezil was statistically significant. As there was no evidence from the muscle tetanic force recordings that any slight increase in synaptic depression was functionally significant, we considered that further use of mice for additional EPP recordings was not justified.

## 4 DISCUSSION

Donepezil, a piperidine anti‐AChE, is widely prescribed for treatment of AD, but relatively little consideration has been given to its effects on peripheral cholinergic synaptic function. Several clinical case studies have reported complications when administration of neuromuscular blockers had been required in patients who were already being treated with donepezil (Baruah et al., 2008; Bhardwaj et al., 2011; Jang et al., 2020). In addition, there have been proposals to extend administration of donepezil, for example, to elderly patients presenting for orthopaedic surgery, in order to mitigate postoperative cognitive dysfunction (Zhu et al., 2021). Studies that underscore how circulating levels of donepezil may affect neuromuscular function are therefore important.

The recommended doses of donepezil prescribed for patients diagnosed with AD are 5–10 mg·day^−1^, given orally, although doses up to 20 mg·day^−1^ are tolerated (Doody et al., 2008). Measurements following single doses of 6.0 mg donepezil resulted in a transient increase of blood plasma concentration to about 15 ng·ml^−1^ (approximately 40 nM) within 30 min, which declined steadily over a period of 144 h to about half this level (Rogers & Friedhoff, 1998). In the steady state, following multiple doses of donepezil (5 mg·day^−1^ for 21 days), plasma concentration reach 17–31 ng·ml^−1^ (45–82 nM), which is within the range of efficacy at mouse NMJs reported here (Rogers et al., 1998). Our findings indicate more than 50% inhibition of muscle AChE by donepezil at concentrations over 50 nM (19 ng·ml^−1^) and substantive effects on the time course of ACh transmitter‐induced endplate depolarisation and neuromuscular function at concentrations of donepezil above 100 nM (38 ng·ml^−1^). We may perhaps estimate that a daily dose of 20 mg donepezil could lead to steady state plasma concentrations as high as 80 ng·ml^−1^ (approximately 200 nM), sufficient to substantially reduce AChE activity in both brain and muscle (see Figure 1). Thus, based on the physiological consequences of in vitro administration to mouse NMJs reported here, doses of donepezil giving rise to the range of plasma concentrations found in humans could account for the range of neuromuscular side effects described in clinical reports. These would seem to be especially likely with relatively high therapeutic doses of donepezil (10–20 mg·day^−1^).

The present findings extend previous reports that were based on more limited data (Clark et al., 2002; Kosasa, Kuriya, & Yamanishi, 1999; Lin et al., 1996, 1997). First, as expected, we confirmed that donepezil inhibits AChE in skeletal muscle. The data indicated an IC_50_ for donepezil of about 30–40 nM for AChE in mouse hind limb muscle homogenates, 2–4 times less effective than for AChE activity in mouse brain homogenates. This may arise because AChE isoforms, varying in size and quaternary structure, are expressed differently in brain and muscle. A predominantly globular (G_4_) form of the enzyme is expressed intracellularly in brain whereas an asymmetric form with a collagen tail (A_12_) is predominantly expressed in the synaptic basal lamina at NMJs (Brimijoin, 1983; Colović et al., 2013). Second, normal neuromuscular function was impaired by donepezil, as indicated by generation of prolonged aftercontractions following brief stimulation at moderate frequencies. Third, donepezil prolonged synaptic depolarisations (MEPPs and EPPs). The incidence of gMEPPs, some evidently triggering spontaneous muscle contractions, was also increased. Overall, however, presynaptic effects of donepezil appeared not to be functionally significant: Neither spontaneous MEPP amplitude or frequency, nor the quantal content of nerve‐evoked EPPs were substantively affected by concentrations of donepezil up to 1 μM. However, definitive confirmation may require voltage‐clamp analysis of endplate currents (EPCs) at NMJs, as the recommended corrections for non‐linear summation of EPPs may not be sufficiently accurate following inhibition of AChE when applied to recordings short muscle fibres, such as the FDB (McLachlan & Martin, 1981). Nevertheless, even if there were changes in evoked release at NMJs exposed to donepezil, as reported in high concentrations of neostigmine, for example (Braga et al., 1993), these would appear to be unlikely to be of such magnitude as to exacerbate any pathophysiological consequences of ACh release when muscle AChE is inhibited (Dissanayake, Margetiny, et al., 2021).

We also found that MEPP amplitude and frequency were unaffected by donepezil, in contrast to a previous report (Lin et al., 1997). However, we confirmed that the incidence of gMEPPs, defined here as spontaneous depolarisations exceeding the median MEPP amplitude by 2.5‐fold or more, was significantly increased after adding donepezil. Similar increases in frequency of gMEPPs occur in NMJs exposed to other anti‐AChEs (Carlson & Dettbarn, 1983). The direct functional significance of gMEPPs is that if their amplitudes reach or exceed the action potential firing threshold in muscle fibres, the result would be spontaneous action potential firing and associated muscle fasciculation (Carlson & Dettbarn, 1983; Gundersen, 1990), which could contribute to some of the symptoms reported by patients taking donepezil (Birks & Harvey, 2006). The mechanism and causes of gMEPPs have been discussed in several previous studies (Gundersen, 1990) but have never been satisfactorily resolved. Insofar as gMEPPs represent release of neurotransmitter from presynaptic terminals, then the effect of donepezil in inducing them implies a presynaptic effect as a proximate cause. Ultimately, however, they could be triggered or facilitated by postsynaptic effects of donepezil, consequential to inhibition of junctional AChE: for instance, a tonic sustained efflux of K^+^ that might depolarise nerve terminals. However, this explanation would predict that MEPP frequency overall should also be increased after adding donepezil and we found no compelling evidence that this was the case.

In addition to reversing neuromuscular block, a notable functional effect of donepezil was the generation of strong aftercontractions accompanying tetanic nerve stimulation. Preliminary data showed an association of localised endplate contractions with increases followed by slow decay of intracellular Ca^2+^ at motor endplates. Localised endplate contractions and aftercontractions, with implied increases in endplate Ca^2+^, have been reported in several previous studies of the effects of carbamate, organophosphorus and piperidine anti‐AChEs but have not been shown directly (Burd & Ferry, 1987; Dissanayake, Chou, et al., 2021; Ferry & Cullen, 1991; Hong & Chang, 1993). Taken together with these previous reports, the present findings support the conclusion that endplate contraction and associated increases in intracellular Ca^2+^ are general consequences of AChE inhibition, rather than off‐target effects of specific anti‐AChE compounds. The molecular mechanism of prolonged endplate Ca^2+^ transients following AChE inhibition is unclear, but a likely pathway is enhanced and protracted transmembrane ionic flux via nAChRs located in the crests of the junctional folds of motor endplates (Slater et al., 1992; Villarroel & Sakmann, 1996). For instance, one of us has also observed localised contractions of NMJs in response to ionophoretically applied ACh to motor endplates in enzymically dissociated, voltage‐clamped mouse FDB muscle fibres, from which basal lamina and AChE had been stripped (RR Ribchester, unpublished data). The Ca^2+^ permeability of junctional nAChRs represents about 4% of their total cationic permeability in adult rodent NMJs but almost twice as much, about 7%, at human NMJs (Fucile et al., 2006; Ragozzino et al., 1998; Villarroel & Sakmann, 1996). We may therefore predict that human NMJs would be more susceptible to endplate contraction after inhibition of AChE and its consequences, than their rodent counterparts. The possibility that other sources or mediators of prolonged endplate contractions, including voltage‐sensitive or Ca^2+^‐sensitive Ca^2+^ channels in plasma membranes or sarcoplasmic reticulum, might contribute to a disproportionate rise in intracellular Ca^2+^, perhaps triggered by Ca^2+^ flux through nAChRs, would be a potentially interesting subject for further experimental investigation. We found in a recent study that endplate contractions and prolonged Ca^2+^ transients (‘calcium bombs’) induced by an organophosphorus anti‐AChE were mitigated by modest (1–4 mM) increases in extracellular Mg^2+^ ionic concentration, both in vivo and in vitro (Dissanayake, Chou, et al., 2021). Further investigation may indicate whether prophylactic or therapeutic administration of MgSO_4_, as utilised, for example, in the treatment of eclampsia (Duley et al., 2003), might also mitigate muscle cramps reported as a side effect elderly patients on standard medication with donepezil (Birks & Harvey, 2006), or the requirement for higher doses of non‐depolarising neuromuscular blockers in such patients when muscle relaxation may be required during surgery (Baruah et al., 2008; Bhardwaj et al., 2011; Jang et al., 2020).

Notwithstanding the overall clarification of the effects of donepezil at NMJs suggested by the present data, there are perhaps a few points that may benefit from further investigation. For instance, there was notable variability in the responses of nerve‐muscle preparations both within and between mice. For each concentration of donepezil, 0–2 female mice were used and between 2 and 5 male mice. These numbers were not sufficient for statistical analysis of the null hypothesis that male and female mice do not differ in their sensitivity to donepezil. The mice, although all sexually mature, also varied in age between 1 and 12 months. In mitigation, there are no studies to our knowledge demonstrating sexual dimorphism of AChE at mouse NMJs, nor on sensitivity to AChE inhibitors, or EPP/EPC characteristics including amplitude, time course or quantal content. The ages of the mice used in the present study all fell within the range of healthy adults, whereas senescent changes in NMJ function generally occur in mice older than 18 months (Kelly & Robbins, 1983) (see also https://www.jax.org/news-and-insights/jax-blog/2017/november/when-are-mice-considered-old). It therefore seems more likely that, as in humans, other genetic, environmental or stochastic factors account for the variable mechanical and electrophysiological responses evident in the data. Nevertheless, it could be worthwhile to extend the findings of the present study to evaluation of potential sex differences or aging on sensitivity of NMJs to donepezil, or to other piperidine anti‐AChE compounds that may be prescribed for treatment of dementias. It would also be useful to validate the present findings by comparing the responses of intact NMJs in isolated human tissue explants (motor‐point biopsies) to those we report here on isolated mouse NMJs. Further studies might also consider potential long‐term consequences of donepezil therapy on the structural and functional integrity of NMJs or other peripheral cholinergic synapses.

In conclusion, we have found that therapeutic concentrations of donepezil significantly inhibit murine muscle AChE and antagonise partial neuromuscular block. Under conditions of high‐frequency repetitive or tetanic excitation of motor nerve axons, donepezil gives rise to long‐lasting contractions of the endplate region and preliminary data have associated these with prolonged focal increases in endplate Ca^2+^. At concentrations in the range 100 nM to 1 μM, donepezil significantly prolongs endplate depolarisation during neuromuscular transmission and increases the incidence of spontaneous gMEPPs. Some of these are of suprathreshold amplitude, capable of giving rise to spontaneous muscle action potentials. Viewed in the context of similar effects of other anticholinesterases, administration of Mg^2+^ could mitigate the neuromuscular side effects of donepezil, including potential adverse effects associated with muscle cramps or requirements for higher concentrations of neuromuscular blockers during surgery.

## CONFLICTS OF INTEREST

None of the authors has any conflict of interests.

## AUTHOR CONTRIBUTIONS

RR Redman (first author) performed experiments, and analysed and reported data; HM performed experiments, and analysed and reported data; KND supervised the research and assisted with experiments; ME contributed to experimental design and obtained funding; RR Ribchester (corresponding author) conceived and designed the study, obtained funding, directed and supervised the research, performed experiments, analysed data and wrote the paper.

## DECLARATION OF TRANSPARENCY AND SCIENTIFIC RIGOUR

This Declaration acknowledges that this paper adheres to the principles for transparent reporting and scientific rigour of preclinical research as stated in the *BJP* guidelines for Design and Analysis, and Animal Experimentation, and as recommended by funding agencies, publishers and other organisations engaged with supporting research.

## Supporting information


**Table S1.** Donepezil increases incidence of gMEPPs. The data indicate the number of muscle fibres expressing at least one gMEPP, compared with numbers of muscle fibres showing no gMEPPs in 30 s of recording. Chi‐square trend test on first two rows: χ^2^(df) = 13.22 (1); P < .05

**Video S1.** Donepezil induces conspicuous and enduring increases in endplate Ca^2+^. Continuous recording at 100 fps of the endplate region of an FDB muscle fibre loaded with the Ca^2+^ indicator Fluo4, showing responses to tetanic stimulation of the tibial nerve supply for 2 s at 20 Hz. The steep localised increase in Ca^2+^/Fluo‐4 fluorescence persisted for several hundred milliseconds beyond the period of tetanic stimulation.

**Figure S1.** Inhibition of muscle AChE activity by neostigmine. Each point represents the mean of triplicate measurements from homogenate of one hindlimb muscle preparation, showing thiocholine production as a measure of AChE enzymic activity, with increasing concentrations of neostigmine. The IC50 was estimated to be about 10–20 nM based on the non‐linear sigmoidal best fit curve (black).

**Figure S2.** Donepezil‐induced aftercontractions are associated with increases in endplate Ca^2+^. **A:** Individual video frames (see Supplementary Video 1) captured during imaging of the endplate region of an FDB muscle fibre loaded with the Ca^2+^ indicator Fluo‐4 (see Methods), during and after tetanic stimulation of the tibial nerve supply. The location of the endplate was confirmed by inspection of individual video frames in Supplementary Video 1. Yellow arrow indicates the location of the motor endplate and cyan arrow indicates the extrajunctional region of the muscle fibre from which Fluo‐4 signals were analysed. Imaging in this region commenced approximately one hour after incubating the preparation in 1 μM donepezil. Muscle action potentials were blocked by pre‐incubation in μCTXGIIIB. Analysis was carried out after digital alignment of successive video frames to compensate for lateral movement during stimulation (see Methods): a – before stimulation; b – approximately 100 ms into the tetanus; c – approximately 100 ms before the end of 2 s stimulation; d – approximately 100 ms after the end of the stimulus train; e – approximately 500 ms after the end of stimulation; f‐ approximately 1 s after tetanic stimulation. **B:** Plots of junctional (red trace; yellow arrow), and extrajunctional (blue trace; cyan arrow) regions of interest corresponding to regions indicated in A. Viewed in association with Supplementary Video 1, the changes in fluorescence (ΔF/F_0_) provide a quantitative indication of the onset and persistence of localised endplate fluorescence compared with extrajunctional fluorescence. The approximate timing of images shown in A are indicated by corresponding letters a‐f.

**Figure S3.** Donepezil induced weak spontaneous muscle contractions in some muscles, consistent with some spontaneous gMEPPs achieving sub‐threshold depolarisation (see Figure. Examples of weak spontaneous twitching of isolated FDB muscles (no nerve stimulation) following addition of A – 10 nM, B‐ 100 nM, C‐ 1 μM donepezil to the recording chamber.

## Data Availability

The data that support the findings of this study are available from the corresponding author upon reasonable request. Some data may not be made available because of privacy or ethical restrictions.
