# Supplementary material for: Donepezil inhibits neuromuscular junctional acetylcholinesterase and enhances synaptic transmission and function in isolated skeletal muscle
Source: Br J Pharmacol. 2022 Sep 15;179(24):5273–89. doi: 10.1111/bph.15940 (PMC9826304; doi:10.1111/bph.15940)
Supplement: Supplementary file 1 — Table S1. Donepezil increases incidence of gMEPPs. The data indicate the number of muscle fibres expressing at least one gMEPP, compared with numbers of muscle fibres showing no gMEPPs in 30 s of recording. Chi‐square trend test on first two rows: χ2(df) = 13.22 (1); P < .05 [file BPH-179-5273-s005.docx]

**SUPPLEMENTARY TABLE 1**

**Supplementary Table 1 Legend:**

Donepezil increases incidence of gMEPPs. The data indicate the number of muscle fibres expressing at least one gMEPP, compared with numbers of muscle fibres showing no gMEPPs in 30 s of recording. Chi-square trend test on first two rows: χ^2^(df)=13.22 (1); P=0.0003
