# Supplementary material for: Donepezil inhibits neuromuscular junctional acetylcholinesterase and enhances synaptic transmission and function in isolated skeletal muscle
Source: Br J Pharmacol. 2022 Sep 15;179(24):5273–89. doi: 10.1111/bph.15940 (PMC9826304; doi:10.1111/bph.15940)
Supplement: Supplementary file 3 — Figure S1. Inhibition of muscle AChE activity by neostigmine. Each point represents the mean of triplicate measurements from homogenate of one hindlimb muscle preparation, showing thiocholine production as a measure of AChE enzymic activity, with increasing concentrations of neostigmine. The IC50 was estimated to be about 10–20 nM based on the non‐linear sigmoidal best fit curve (black). [file BPH-179-5273-s003.pdf]

Supplementary Figure 1

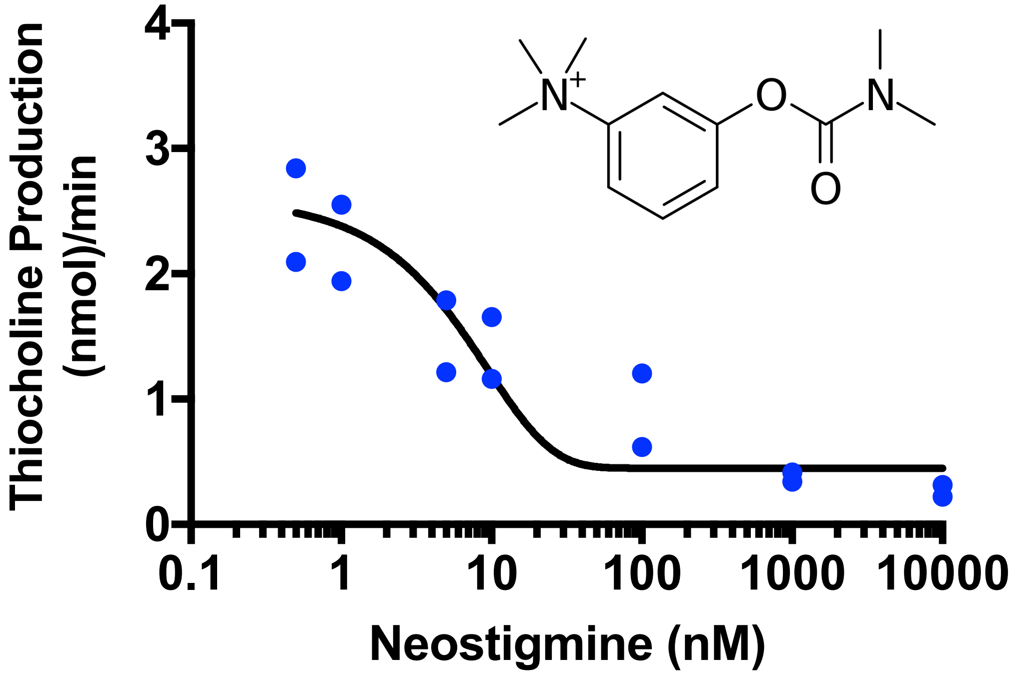

Supplementary Figure 1
Inhibition of muscle AChE activity by neostigmine. Each point represents the mean of triplicate measurements from homogenate of one hindlimb muscle preparation, showing thiocholine production as a measure of AChE enzymic activity, with increasing concentrations of neostigmine. The IC50 was estimated to be about 10-20 nM based on the non-linear sigmoidal best fit curve (black).
