# Supplementary material for: Donepezil inhibits neuromuscular junctional acetylcholinesterase and enhances synaptic transmission and function in isolated skeletal muscle
Source: Br J Pharmacol. 2022 Sep 15;179(24):5273–89. doi: 10.1111/bph.15940 (PMC9826304; doi:10.1111/bph.15940)
Supplement: Supplementary file 4 — Figure S2. Donepezil‐induced aftercontractions are associated with increases in endplate Ca2+. A: Individual video frames (see Supplementary Video 1) captured during imaging of the endplate region of an FDB muscle fibre loaded with the Ca2+ indicator Fluo‐4 (see Methods), during and after tetanic stimulation of the tibial nerve supply. The location of the endplate was confirmed by inspection of individual video frames in Supplementary Video 1. Yellow arrow indicates the location of the motor endplate and cyan arrow indicates the extrajunctional region of the muscle fibre from which Fluo‐4 signals were analysed. Imaging in this region commenced approximately one hour after incubating the preparation in 1 μM donepezil. Muscle action potentials were blocked by pre‐incubation in μCTXGIIIB. Analysis was carried out after digital alignment of successive video frames to compensate for lateral movement during stimulation (see Methods): a – before stimulation; b – approximately 100 ms into the tetanus; c – approximately 100 ms before the end of 2 s stimulation; d – approximately 100 ms after the end of the stimulus train; e – approximately 500 ms after the end of stimulation; f‐ approximately 1 s after tetanic stimulation. B: Plots of junctional (red trace; yellow arrow), and extrajunctional (blue trace; cyan arrow) regions of interest corresponding to regions indicated in A. Viewed in association with Supplementary Video 1, the changes in fluorescence (ΔF/F0) provide a quantitative indication of the onset and persistence of localised endplate fluorescence compared with extrajunctional fluorescence. The approximate timing of images shown in A are indicated by corresponding letters a‐f. [file BPH-179-5273-s004.pdf]

Supplementary Figure 2

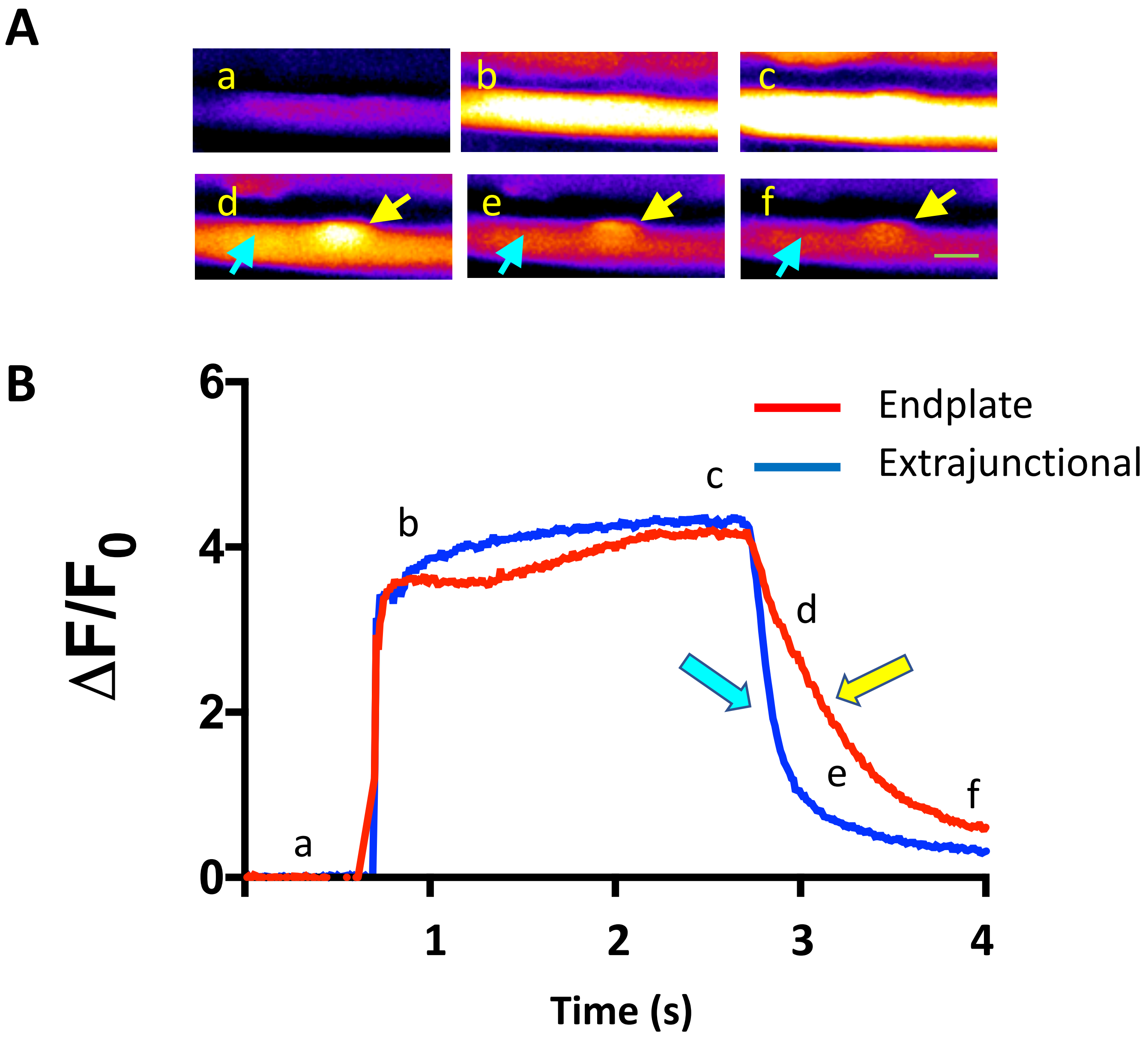

Supplementary **Figure 2**
Donepezil-induced aftercontractions are associated with increases in endplate Ca^{2+} .
A: Individual video frames (see Supplementary Video1) captured during imaging of the endplate region of an FDB muscle fibre loaded with the Ca^{2+} indicator Fluo-4 (see Methods), during and after tetanic stimulation of the tibial nerve supply. The location of the endplate was confirmed by inspection of individual video frames in Supplementary Video 1. Yellow arrow indicates the location of the motor endplate and cyan arrow indicates the extrajunctional region of the muscle fibre from which Fluo-4 signals were analysed.
Imaging in this region commenced approximately one hour after incubating the preparation in 1 µM donepezil. Muscle action potentials were blocked by pre-incubation in µCTXGIIIB. Analysis was carried out after digital alignment of successive video frames to compensate for lateral movement during stimulation (see Methods): a – before stimulation; b – approximately 100 ms into the tetanus; c – approximately 100 ms before the end of 2s stimulation; d – approximately 100 ms after the end of the stimulus train; e – approximately 500 ms after the end of stimulation; f- approximately 1s after tetanic stimulation.

B: Plots of junctional (red trace; yellow arrow), and extrajunctional (blue trace; cyan arrow) regions of interest corresponding to regions indicated in A. Viewed in association with Supplementary Video 1, the changes in fluorescence ($\Delta F/F_0$) provide a quantitative indication of the onset and persistence of localised endplate fluorescence compared with extrajunctional fluorescence. The approximate timing of images shown in A are indicated by corresponding letters a-f.
