# Supplementary material for: Donepezil inhibits neuromuscular junctional acetylcholinesterase and enhances synaptic transmission and function in isolated skeletal muscle
Source: Br J Pharmacol. 2022 Sep 15;179(24):5273–89. doi: 10.1111/bph.15940 (PMC9826304; doi:10.1111/bph.15940)
Supplement: Supplementary file 5 — Figure S3. Donepezil induced weak spontaneous muscle contractions in some muscles, consistent with some spontaneous gMEPPs achieving sub‐threshold depolarisation (see Figure. Examples of weak spontaneous twitching of isolated FDB muscles (no nerve stimulation) following addition of A – 10 nM, B‐ 100 nM, C‐ 1 μM donepezil to the recording chamber. [file BPH-179-5273-s001.pdf]

Supplementary Figure 3

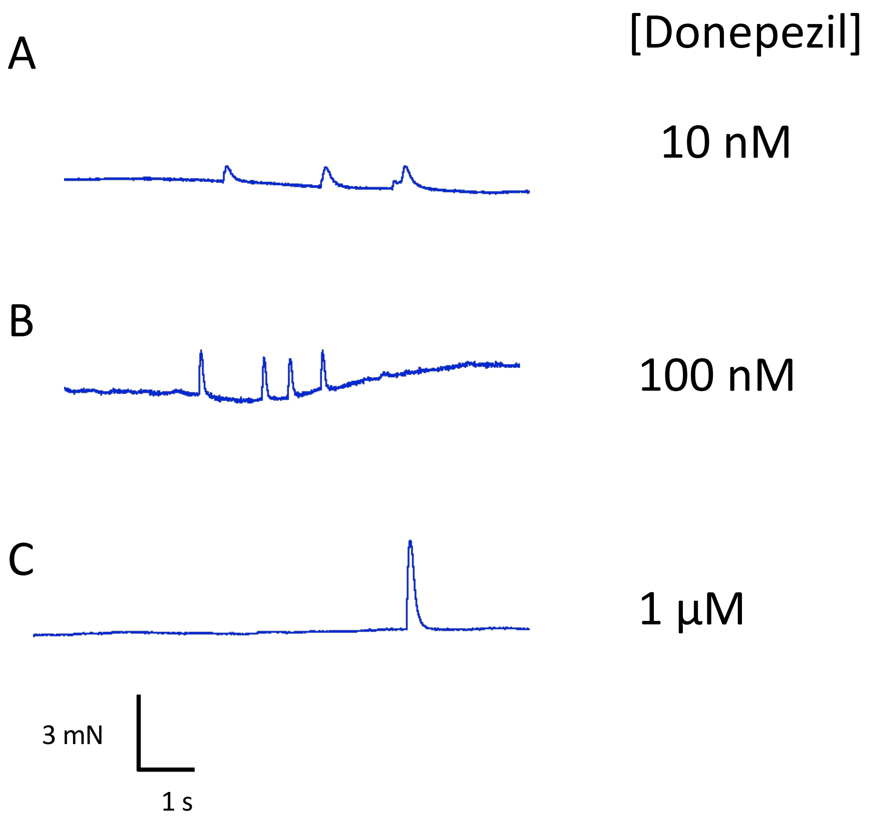

Supplementary Figure 3
Donepezil induced weak spontaneous muscle contractions in some muscles, consistent with some spontaneous gMEPPs achieving sub-threshold depolarisation (see Figure . Examples of weak spontaneous twitching of isolated FDB muscles (no nerve stimulation) following addition of A – 10 nM, B- 100 nM, C- 1 μM donepezil to the recording chamber.
